# Bevacizumab Improves Quality of Life in Patients with Recurrent Glioblastoma

**DOI:** 10.1155/2011/602812

**Published:** 2011-10-02

**Authors:** Seema Nagpal, Griffith Harsh, Lawrence Recht

**Affiliations:** ^1^Department of Neurology and Neurological Sciences, Stanford University School of Medicine, Stanford, CA 94305, USA; ^2^Advanced Medicine Center, 875 Blake Wilbur Drive, CC2221 Stanford, CA 94305, USA; ^3^Department of Neurosurgery, Stanford University School of Medicine, Stanford, CA 94305, USA

## Abstract

*Objective*. To quantify the benefits in survival and quality of life in patients receiving bevacizumab (BEV) for recurrent glioblastoma (GBM). *Methods*. This is a retrospective study of 40 adult patients with recurrent GBM treated between 2005 and 2009 at a single institution. All patients had initial treatment with surgery, radiation, and concurrent temozolomide, then monthly temozolomide. Over 250 charts were screened. Sufficient data was available for 20 patients treated with BEV and 20 patients who did not receive BEV at the time of recurrence. The independent living score (ILS), designed to reward long-term independent survival, was calculated for each patient. *Results*. The mean ILS was nearly double in the BEV group compared to the No-BEV group (15.0 versus 8.2, *P* = 0.002, *t*-test). Two months after initiation of therapy, the median steroid dose dropped by over 90% in patients treated with BEV, but doubled in the NoBEV group. Median survival from the time of recurrence was significantly affected: 10.6 months in the BEV group versus 4.2 months (*P* < 0.001, log rank survival) in the NoBEV group. *Conclusions*. BEV increases independent living and lengthens overall survival after GBM recurrence. Reduction in steroid dose may contribute to prolonged independence.

## 1. Introduction

In May of 2009, the FDA approved the use of bevacizumab (BEV) for recurrent glioblastoma (GBM) based on imaging and clinical responses demonstrated in Phase II clinical trials [1–6]. Many centers, particularly in the USA, have rapidly adopted bevacizumab into regular clinical practice, though there is no phase III evidence of its efficacy. Some propose that the FDA rescinds approval for BEV if current randomized studies do not show efficacy [7–10]. Others strongly believe that it is an effective, potent drug despite the modest response rate and effect on progression-free survival demonstrated in previous trials. We hypothesized that BEV both lengthens patient survival and improves “quality of life,” which is difficult to study clinically. 

Several years ago, we proposed an Independent Living Score (ILS) in which the patient's capacity to remain independent is used as a global measure of quality of life [11]. The ILS is based on the Karnofsky performance score and is weighted to increase the score of patients who retain independence later in their course. We tested our hypothesis that patients who receive BEV live longer and remain independent longer than those who do not receive BEV, by comparing a cohort of patients who received BEV with a contemporaneous cohort whose members, for insurance reasons, did not receive BEV, using overall survival and ILS as endpoints. In many cases, patients with public insurance received BEV after appeal, while private insurers declined to cover BEV prior to FDA approval.

## 2. Methods

Permission was granted from Stanford's IRB to perform this study. Patients were identified from the senior author's (L. Recht) personal database that tracks all patients at Stanford Medical Center with primary brain tumors. Hospital charts were screened to identify adult patients (age >18) fulfilling the following requirements: (i) diagnosis of glioblastoma or gliosarcoma without prior diagnosis of lower grade glioma; (ii) upfront treatment with six weeks of concurrent radiotherapy and temozolomide; (iii) MRI evidence of tumor recurrence; (iv) at least three Stanford encounters over the patient's course; (v) recording of KPS at each visit; (vi) administration of at least one IV infusion of bevacizumab (BEV group). More than 200 charts were screened. Twenty patients meeting these criteria were identified. Our intention was to create a control group 2-3 times larger than the BEV group, but sufficient data was available for only twenty patients meeting all criteria except vi (No-BEV group).

All BEV patients were treated with the same regimen of BEV 7.5–10 mg/kg every two weeks for three infusions followed by continued infusions at three-week intervals. Seven patients initially received BEV alone; 13 others received BEV initially in combination with other chemotherapeutic agents: irinotecan (*n* = 6), lomustine (*n* = 4), and temozolomide (*n* = 3) or no additional therapy (*n* = 7). Patients in the No-BEV group received lomustine (*n* = 4), lomustine with erlotinib (*n* = 1), BCNU (*n* = 1), temozolomide (*n* = 2), short-course additional radiation with temozolomide (*n* = 1), cyclophosphamide with erlotinib (*n* = 1), reoperation followed by BCNU (*n* = 1), experimental chemotherapy (*n* = 1), or no additional therapy at the time of recurrence (*n* = 8). 

 Imaging, pathology reports, and clinic notes were reviewed for all patients. All 40 patients were seen by one physician (L. Recht). The KPS, assigned at the time of visit, not retrospectively, was recorded. The ILS was calculated as described by Recht et al. [11] (example calculations available in the appendix of Recht's paper). Patients received 2 points for KPS ≥ 70, 1 point for KPS ≥ 60, and no points for a score of 50 or less. In cases where more than one KPS was available in a month, the lower of the two point scores was used to calculate ILS (see Table 1). The point score for each month was then multiplied by a factor emphasizing independence late in a patient's course: the number of months since diagnosis divided by the overall survival. These weighted monthly point scores were then summed to yield an individual patient's ILS. Modified Macdonald criteria [12] defined initial recurrence. The determination of progression despite BEV was made according to RANO working group criteria [13] of MRI and clinical findings. Statistical analysis was performed using a SigmaStat package. 

## 3. Results

**Figure 1:**
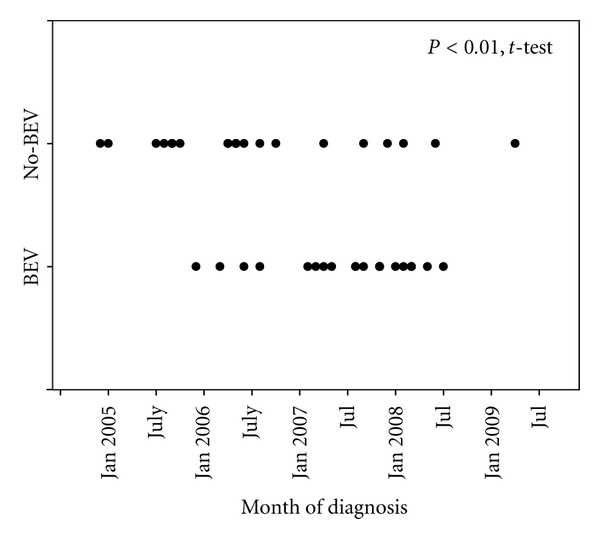
Month of diagnosis of patient cohorts. Time line of patient accrual to BEV and No-BEV groups as a function of month of GBM diagnosis. Dot plot indicates month of diagnosis. Using 1/1/2005 as Time 0, there is a statistically but not clinically significant difference in dates of diagnosis.

Prior to disease recurrence, the two groups were comparable in terms of age, initial KPS, treatment, and time to recurrence (Table 2). All patients were diagnosed in the interval between 2005 and 2009. Considering 1/1/2005 as time 0, there was a statistically significant difference between BEV and No-BEV groups in terms of the dates of diagnosis (36 ± 8.7 versus 26.0 ± 14.1 months after 1/1/2005, *P* < 0.01, *t*-test) (Figure 1). However, this was unlikely to be clinically significant. The mean times of diagnosis for the BEV and No-BEV groups were December 2009 and February 2009, respectively. This 10-month difference in diagnosis date likely altered clinical care only in respect to use of BEV, which became universally available to patients with recurrent GBM in May 2009. 

BEV patients received a mean of 10.1 ± 4.8 infusions (range: 2–20). Complications definitely or probably attributable to BEV occurred in eight patients (40%) and included DVT (2, both grade 3), asymptomatic grade 1 intracranial hemorrhage (2), grade 1 hypertension, diverticulitis leading to death (grade 4), a grade 2 wound-healing problem, and grade 2 allergy (1 each). One of the two patients with steroid myopathy (causing difficulty walking unassisted) prior to BEV regained full strength after initiation of BEV and reduction of steroid.

Six patients (30%) in the No-BEV group had complications: DVT (1), pulmonary embolism that ultimately led to death (1), steroid myopathy (3), cytomegalovirus sepsis leading to death in a patient on high-dose dexamethasone alone (1), myelosuppression in patients on high-dose dexamethasone alone (2), and myelosuppression in patients on other chemotherapy (1). 

**Figure 2:**
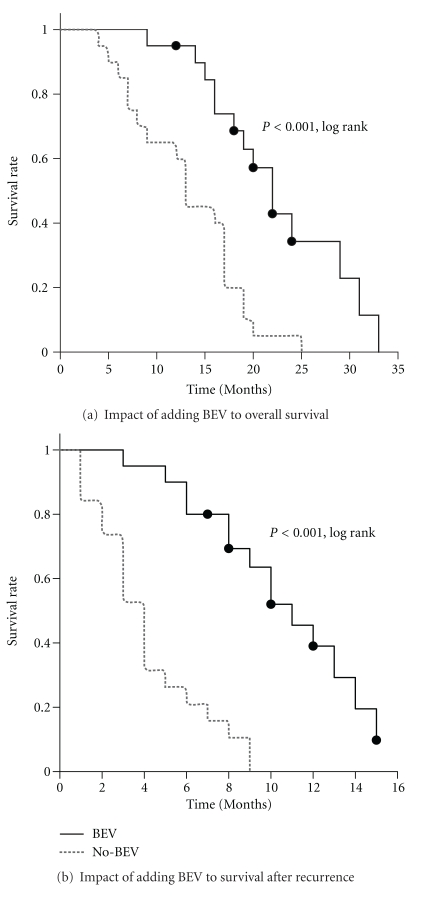
BEV increases survival when administered to patients with recurrent GBM. (a) Overall survival. (b) Survival after recurrence. BEV (solid line), No-BEV (dotted line).

Using conventional measures, the PFS6 for the BEV treated groups was 45% and the mean PFS was 5.25 ± 3.3 months. This does not significantly differ from the survival postrecurrence of the No-BEV cohort (PFS 3.95 ± 2.7 months, P, NS, *t*-test). However, using more global outcome scales, the administration of BEV resulted in a significant improvement in outcome, including a robust increase in both total median survival [22.7 (19.3–26.1, 95% CI) versus 13.2 (10.6–15.8) months, *P* < 0.001, log rank survival] and median survival from time of recurrence [10.6 (8.9–12.3) versus 4.2 (3.0–5.3) months, *P* < 0.001, log rank survival] (Figure 2).

 To reflect overall quality of life, we utilized the independent living score (ILS), which quantifies patient independence and has been validated in a GBM-afflicted population. The scale is weighted to reward sustained independence and penalize prolonged periods of nonindependence (for more details, see Recht et al. [11]). Using this measure, we noted a marked superiority in ILS for the BEV group relative to the No-BEV group (15.0 ± 6.5 versus 8.2 ± 6.0, *P* = 0.002, *t*-test) (Figure 4). Longer survival in the BEV group was accompanied by robust improvement in ILS, indicating that the survival of these patients was marked by retained independence.

**Figure 3:**
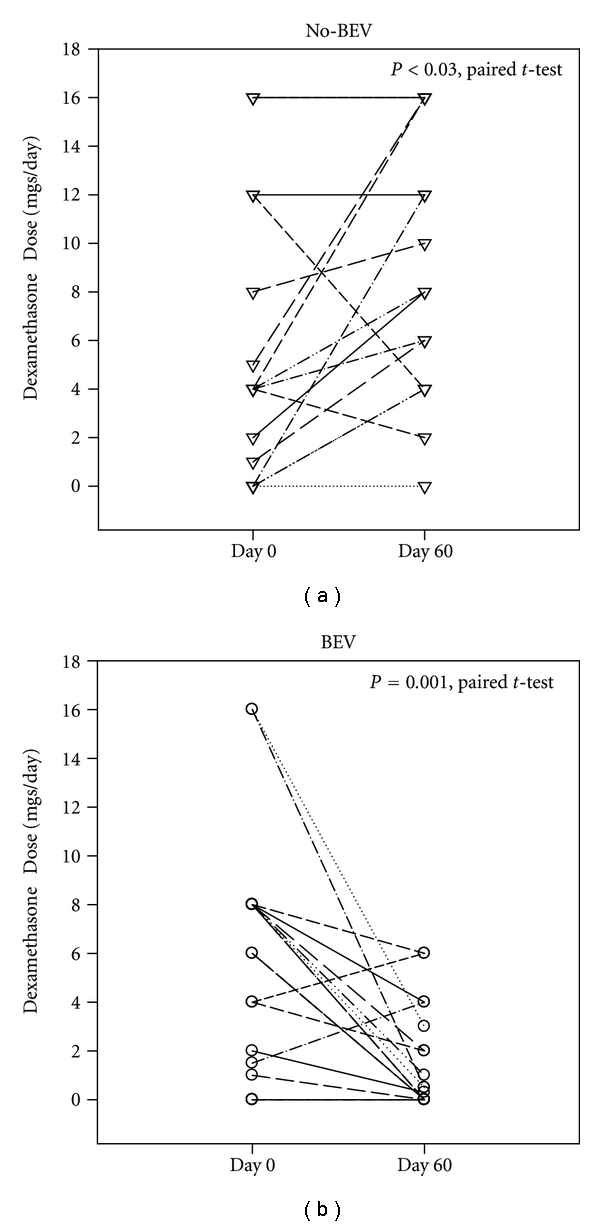
Impact of BEV on steroid requirement. Steroid dose is markedly decreased after BEV administration. Paired doses of dexamethasone at initiation and two months after initiation of BEV or recurrence in the No-BEV group demonstrate a marked difference in ability to reduce steroid dosage.

**Figure 4:**
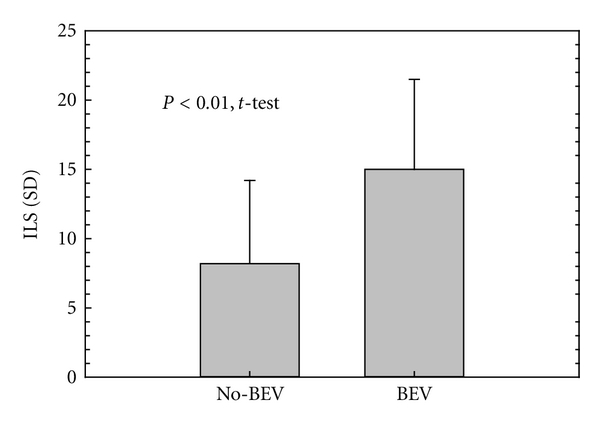
Impact of BEV in the independent living score. Time of functional independence is increased after BEV. The mean Independent Living Score (ILS) in patients treated with BEV compared to those in the No-BEV group is nearly double. Results are depicted as mean ± SD.

The administration of BEV also dramatically decreased steroid usage. While median dexamethasone dose was not significantly different between the two groups at the time of recurrence (6 mgs versus 4 mgs, BEV versus No-BEV, P, NS), by two months after the initiation of BEV infusions, the median dexamethasone dose had decreased to 0.4 mgs in the BEV group. At the corresponding time point in the No-BEV group, it had increased to 8 mgs (*P* < 0.001, rank sum test) (Figure 3).

## 4. Discussion

Unlike other expensive, approved therapies, such as the carmustine polymer wafer (Gliadel) [14], the introduction of BEV has dramatically changed practice patterns in the United States, even in the absence of Phase III evidence. Physicians who use BEV believe it to be effective and useful. However, BEV's complication rates, its tendency to produce tumor dispersion/invasion [15–17], poor patient survival after progression of BEV-treated tumors [18], and BEV's minimal impact on standard surrogate markers of GBM outcome, such as PFS [3–5, 15, 19, 20], support the counterargument that BEV's overall benefit does not justify its routine use. 

Our data demonstrate a robust improvement in patient independence, in combination with an increase in overall survival, in patients who receive BEV. These data help explain the enthusiasm for BEV despite unimpressive clinical trial data. Using surrogate markers such as PFS, we observed a PFS6 and median duration of survival similar to those reported in the literature [3–5, 15, 19–24]. However, even with a BEV complication rate of 40%, there is a dramatic shift in the entire survival curve “to the right,” with a survival advantage for BEV-treated patients approaching 9 months from time of recurrence (a duration exceeding 150% of that experienced by patients not receiving BEV). This improvement in survival duration is accompanied by a dramatic increase in quality of life, at least as measured in regard to the very important consideration of maintained patient independence. 

There are several implications of our results. First, the effect of BEV is fundamentally different from that of other standard antiglioma treatments. There is an extremely high rate of clinical response when using ILS and overall survival as endpoints, rather than PFS or PFS6. Additionally, there is an impressive increase in area under the survival curve after recurrence, which means that the bulk of patients receiving BEV are getting a survival benefit of 5-6 months. In contrast, the EORTC study that added temozolomide to radiation therapy increased the median overall survival only a modest 6 weeks and was universally adopted primarily because of its “tail” effect: an increase in the number of long-term 2-year survivors from 8 to 26% [25]. Our results with BEV (Figure 2), which seem to be in general agreement with the observations of others, do not demonstrate this “tail” effect. Patients are still dying within the expected time range. However, almost all patients live longer. Few patients have dramatically lengthened survivals on BEV and none are “cured.” 

The high ILS scores also imply that the time gained with BEV is of high quality. We believe that this is an important aspect of the “Avastin effect" which has, paradoxically, both confounded analysis and motivated clinicians to use it. Measures such as PFS and PFS6 inadequately assess BEV's effect because patients often remain clinically stable despite tumor progression on MRI. We believe that the quality of a patient's survival is often a more meaningful measure of the success of a therapy than is MRI. Functional independence is both strongly influenced by BEV and highly valued by our patients and their families.

This improvement in quality of life with BEV may be predominantly attributable to the reduction of steroid dose it permits. For over 50 years, steroids, primarily dexamethasone, have been used routinely to treat peritumoral brain edema [26]. Although they are highly effective against chronic edema, steroids given for prolonged periods produce many side effects, including myopathy, diabetes, and infection, which can markedly diminish a patient's quality of life and duration of survival. Thus, standard practice is to use as low a dose as possible. 

Although not initially recognized, one of the most compelling benefits of BEV administration is its reduction of peritumoral edema and the need for steroids. While this statement echoes the criticism that the majority of BEV's clinical effect is that of a “very powerful steroid” [17, 27–29], our observations suggest that this should not be interpreted unfavorably. The average addition of over seven months of high-quality survival after GBM recurrence, compares well with that achieved with any cytotoxic agent. 

The retrospective nature and lack of a control group at least double the size of the index cohort are major limitations of this study. However, several aspects of this study help control for unintentional biases. The two patient cohorts were not intentionally case matched, but they are well balanced in terms of histology, age, KPS, time to first recurrence, and the use of additional chemotherapy at the time of recurrence. All patients were accrued in the “modern era” of GBM therapeutics, after the universal adoption of concurrent upfront radiation with temozolomide [25], with only a 10-month difference in the mean date of diagnoses between the two groups. With the exception of BEV approval, there were no changes in clinical practice during this time, implying that the primary difference in treatment received by these two groups was insurance approval of BEV. Many of our public health program patients had access to BEV in advance of privately insured patients. Thus, the confounding of conclusion by historical control comparisons [30] was limited in this study.

This study further supports the use of BEV in patients with recurrent GBM, not only for prolongation of survival, but maintenance of independence, as well. Our data shed no light on the most advantageous timing of initiating BEV, a question that is currently being addressed in Phase III studies. However, our data do suggest that the use of traditional radiologic endpoints in trials for recurrent GBM may underestimate the effects of experimental agents.

## Figures and Tables

**Table 1:** Assignment of the independence score based on Karnofsky performance score.

Score (%)	Criteria	Independence score
100	Normal. No complaints or evidence of disease.	Able to carry on near-normal activity and to work; no special care needed. *Independence Score: 2 *
90	Able to carry on activity, minor signs of disease.	
80	Normal activity with effort, some signs of disease.	
70	*Cares for self.* Unable to carry on normal activity or active work.	

60	Requires occasional assistance, but cares for personal needs.	Unable to work; able to live at home and care for most personal needs; varying amount of assistance needed. *Independence Score: 1 *
50	Requires considerable assistance and medical care.	

40	Disabled. Requires special care and assistance.	Unable to care for self; requires equivalent of institutional or hospital care; disease may be progressing rapidly. *Independence Score: 0 *
30	Severely disabled. Hospitalization needed, but death not imminent.	
20	Very sick. Hospital admission needed. Active supportive care.	
10	Moribund. Fatal process rapidly progressing	
0	Dead	

**Table 2:** Demographics of patient cohorts.

	No bevacizumab	Bevacizumab	Statistical significance of differences
Histology	GBM 20	GBM 19	Not significant
		Gliosarcoma 1	
Age (years)	55 ± 9.3	58.7 ± 10.7	Not significant
Initial KPS	70 (60–80)	75 (65–85)	Not significant
Time to first recurrence (months)	9.25 ± 4.7	10.5 ± 4.8	Not significant
Mean time to diagnosis (from 1/1/05)	25.9 ± 14.1	36.7 ± 8.8	*P* < 0.01
